# Genomic markers of recurrence risk in atypical meningioma following gross total resection

**DOI:** 10.1093/noajnl/vdad004

**Published:** 2023-01-10

**Authors:** Rachael A Vaubel, Rahul Kumar, Taylor M Weiskittel, Sarah Jenkins, Surendra Dasari, Joon H Uhm, Daniel H Lachance, Paul D Brown, Jamie J Van Gompel, Robert B Jenkins, Benjamin R Kipp, William R Sukov, Caterina Giannini, Derek R Johnson, Aditya Raghunathan

**Affiliations:** Department of Laboratory Medicine and Pathology, Mayo Clinic, Rochester, Minnesota, USA; Department of Neurologic Surgery, Mayo Clinic, Rochester, Minnesota, USA; Molecular Pharmacology and Experimental Therapeutics, Mayo Clinic, Rochester, Minnesota, USA; Department of Quantitative Health Sciences, Mayo Clinic, Rochester, Minnesota, USA; Department of Quantitative Health Sciences, Mayo Clinic, Rochester, Minnesota, USA; Department of Neurology, Mayo Clinic, Rochester, Minnesota, USA; Department of Neurology, Mayo Clinic, Rochester, Minnesota, USA; Department of Radiation Oncology, Mayo Clinic, Rochester, Minnesota, USA; Department of Neurologic Surgery, Mayo Clinic, Rochester, Minnesota, USA; Department of Laboratory Medicine and Pathology, Mayo Clinic, Rochester, Minnesota, USA; Department of Laboratory Medicine and Pathology, Mayo Clinic, Rochester, Minnesota, USA; Department of Laboratory Medicine and Pathology, Mayo Clinic, Rochester, Minnesota, USA; Department of Laboratory Medicine and Pathology, Mayo Clinic, Rochester, Minnesota, USA; Department of Neurology, Mayo Clinic, Rochester, Minnesota, USA; Department of Radiology, Mayo Clinic, Rochester, Minnesota, USA; Department of Laboratory Medicine and Pathology, Mayo Clinic, Rochester, Minnesota, USA

**Keywords:** atypical meningioma, copy number, DNA methylation profiling, gross total resection

## Abstract

**Background:**

Meningiomas are the most common primary central nervous system (CNS) tumor in adults and CNS World Health Organization grade 2 (atypical) meningiomas show an intermediate risk of recurrence/progression. Molecular parameters are needed to better inform management following gross total resection (GTR).

**Methods:**

We performed comprehensive genomic analysis of tumor tissue from 63 patients who underwent radiologically confirmed GTR of a primary grade 2 meningioma, including a CLIA-certified target next-generation sequencing panel (*n* = 61), chromosomal microarray (*n* = 63), genome-wide methylation profiling (*n* = 62), H3K27me3 immunohistochemistry (*n* = 62), and RNA-sequencing (*n* = 19). Genomic features were correlated with long-term clinical outcomes (median follow-up: 10 years) using Cox proportional hazards regression modeling and published molecular prognostic signatures were evaluated.

**Results:**

The presence of specific copy number variants (CNVs), including -1p, -10q, -7p, and -4p, was the strongest predictor of decreased recurrence-free survival (RFS) within our cohort (*P* < .05). *NF2* mutations were frequent (51%) but did not show a significant association with RFS. DNA methylation-based classification assigned tumors to DKFZ Heidelberg benign (52%) or intermediate (47%) meningioma subclasses and was not associated with RFS. H3K27 trimethylation (H3K27me3) was unequivocally lost in 4 tumors, insufficient for RFS analysis. Application of published integrated histologic/molecular grading systems did not improve prediction of recurrence risk over the presence of -1p or -10q alone.

**Conclusions:**

CNVs are strong predictors of RFS in grade 2 meningiomas following GTR. Our study supports incorporation of CNV profiling into clinical evaluation to better guide postoperative patient management, which can be readily implemented using existing, clinically validated technologies.

**Key Points**

- Specific CNVs predict grade 2 meningioma recurrence risk after GTR.
- Risk of atypical meningioma recurrence associated with -1p, -4p, -7p, -10q, and -18.

**Importance of the Study**

Meningiomas are the most common primary central nervous system (CNS) tumors in adults. Although the majority are low grade (CNS World Health Organization [WHO] grade 1) with favorable prognosis, atypical (CNS WHO grade 2) meningiomas show an intermediate risk of recurrence and the role of adjuvant radiation therapy following gross total resection (GTR) remains controversial. Molecular parameters may help better inform management following GTR. Our study demonstrates that specific copy number variants (CNVs), including -1p, -10q, -7p, and -4p, are a strong predictor of decreased recurrence-free survival (RFS) (*P* < .05). In contrast, DNA methylation-based classification was not associated with RFS, nor was the *NF2* mutation status. Our study supports incorporation of CNV profiling into clinical evaluation of atypical meningiomas to better guide postoperative patient management and can be readily implemented using existing, clinically validated technologies.

Meningiomas are common central nervous system (CNS) tumors, representing approximately 45% of primary CNS tumors in patients over age 40.^1^ Within the 2021 World Health Organization (WHO) Classification of CNS tumors, meningiomas are considered to comprise a single entity, assigned a CNS WHO grade 1 (approximately 80%), grade 2 (approximately 20%), or grade 3 (approximately 2%).^2^ Grading of meningiomas remains largely based on morphology, with 2021 WHO Classification incorporating *TERT* promoter mutation and *CDKN2A/B* homozygous deletion as molecular criteria to support a CNS WHO grade 3 designation.^2^ Recent studies uniformly affirm the prognostic significance of WHO grade, but histology alone is inadequate to fully predict recurrence risk. This is particularly true for WHO grade 2 tumors, which have an intermediate recurrence risk and unpredictable clinical course following gross total resection (GTR).

Recent large-scale genomic studies have aimed to identify molecular subgroups of meningioma and molecular markers that better predict recurrence risk. Numerous meningioma classification systems have been proposed based on genomic driver alterations,^3,4^ copy number,^5^ RNA expression,^6,7^ DNA methylation signature,^8–10^ integrated multi-omic signatures,^8,10,11^ or have incorporated both histologic and molecular features.^12,13^ When applied across large cohorts of meningioma patients, these molecular signatures may better predict recurrence risk than WHO grade alone. However, most prior studies included meningiomas of all histologic grades and have not carefully controlled for extent of resection. As optimal treatment of patients following GTR of CNS WHO grade 2 meningiomas remains controversial, improved molecular stratification may be of particular benefit in these patients.

In this study, we performed detailed histologic and genomic analyses of a retrospective cohort of patients with radiographically confirmed GTR of histologically CNS WHO grade 2 meningioma, including chromosomal microarray, targeted next-generation sequencing (NGS), DNA methylation profiling, and H3K27me3 immunohistochemistry (IHC), to evaluate for molecular features associated with risk of tumor recurrence.

## Methods

### Patient Cohort

We previously evaluated the impact of radiation therapy across a cohort of 69 patients who underwent radiographically confirmed GTR of WHO grade 2 meningiomas at our institution between 1988 and 2011.^14^ All tumors were verified to meet histologic criteria for a CNS WHO 2 designation according to the 2021 WHO Classification of CNS Tumors. Tumor tissue from 63 patients of this initial series were available for further clinical and genomic analysis. Medical records were reviewed (through March 15, 2022) for additional follow-up since publication of the original series in 2017.^14^ All aspects of the study were approved by the Institutional Review Board and biospecimens subcommittee.

### Chromosomal Microarray Analysis

DNA was extracted from formalin-fixed paraffin embedded (FFPE) tissue sections using the QIAamp DNA FFPE Tissue Kit (Qiagen) as described previously.^15^ Copy number changes were assessed using a molecular inversion probe array (MIP) (OncoScan CNV Plus Array, Thermo-Fisher Scientific), which covers common copy number changes across 900 cancer genes. Briefly, isolated DNA was hybridized to MIPs; gap filling was performed utilizing the bound FFPE DNA as a template to circulize MIPs; FFPE DNA was removed using an exonuclease; and the circularized MIPs were linearized, amplified, digested, and hybridized to oligonucleotide arrays. The arrays were scanned on a GeneChip Scanner (Affymetrix), and the images processed with OncoScan Console Software (Affymetrix). CNVs and copy neutral loss of heterozygosity were identified by manual annotation of the data using Chromosome Analysis Suite (Affymetrix). Arm level alterations were defined as greater than 50% of the arm gained or lost relative to the copy neutral state and a deviation of >0.3 from baseline.

### Targeted NGS

To characterize known CNS somatic mutations, a custom GeneRead DNAseq Targeted Neuro-Oncology Panel (Qiagen) (Supplementary Table S1) was used to amplify selected targets and the amplicons were purified using Beckman Coulter’s AMPure XP kit. Libraries were prepared using Illumina’s TruSeq kit and sequenced on a HiSeq 2500 (Illumina) using the rapid run mode. Variants with allele frequency ≥ 10% from regions with ≥200X coverage were manually annotated as pathogenic or as a variant of unknown significance.

### DNA Methylation Profiling

CpG methylation analysis was performed using Infinium 850k Methylation array (Illumina) according to the manufacturer’s recommendations. Briefly, 200 ng of DNA extracted from each sample was utilized to restore the degraded FFPE DNA to a state that is analyzable by the Infinium HD FFPE whole genome amplification workflow as described in the Infinium FFPE Restoration guide (Illumina). DNA was denatured using 8 µL of NaOH 0.1N for 10 min at room temperature. A 1 h reaction at 37°C was then performed with Primer Pre-Restore (PPR) and Amplification Mix Restore (AMR) reagents supplied by the kit manufacturer in which DNA repair is accomplished. Restored DNA was cleaned using a ZR-96 DNA Clean and Concentrator-5 kit (Zymo Research) following the manufacturer’s protocol. DNA was concentrated between 150 ng/uL of concentration. Between 100 and 200 ng of prepared DNA was used as input for the hybridization on the Illumina 850K Epic BeadChip array and processed according to kit instructions. Data were quality assessed using Illumina Genome Studio software (Illumina), and idat files were uploaded to the DKFZ Heidelberg Classifier v12.5 (www.molecularneuropathology.org).^16^

### RNA Sequencing

Gene expression analysis was performed using a previously described method.^17^ Briefly, RNA was extracted from the frozen tumor tissue material using the AllPrep DNA/RNA FFPE kit (Qiagen) according to the manufacturer’s recommendations. RNA was converted to cDNA and cDNA concertation and size distribution was measured using Agilent Fragment Analyzer and Qubit fluorometry (Invitrogen) following the manufacturer’s recommendations. Approximately 300 ng of cDNA was utilized to prepare paired-end sequencing libraries using TruSeq RNA Exome kit (Illumina) according to the manufacturer’s recommendations. Concentration and size of the prepared libraries were measured as previously described. Prepared libraries were sequenced at 6 samples per lane following Illumina’s standard protocol using the Illumina cBot and HiSeq 3000/4000 PE Cluster Kit. The flow cells were sequenced as 101 × 2 paired end reads on an Illumina HiSeq 4000 using the HiSeq 3000/4000 sequencing kit and HD 3.4.0.38 collection software. Base-calling was performed using Illumina’s RTA version 2.7.7.

Raw sequencing data was processed using MAPRSeq pipeline to extract gene-wise read count data as previously published.^18^ Briefly, FastQC software was utilized to perform quality assessment of the read data of each sample. Sequenced reads were aligned to the hg38 reference using the STAR aligner.^19^ HTSeq software was utilized to extract gene-wise read count data based on Ensembl version 78 transcriptome.^20^ Analysis of raw counts and figure generation for RNAseq data was completed in R.^21^ Raw counts were normalized and differential expression between progressors and non-progressors was calculated using DESeq2.^22^ Differential expression was visualized using a volcano plot generated using Enhanced Volcano.^23^ Uniform Manifold Approximation and Projection (UMAP) plots were creating using the UMAP (https://CRAN.R-project.org/package=umap) and ggplot2 packages.^24^ Correlation between patient transcriptomes was measured by calculating the Pearson’s correlation between normalized count profiles. The correlation matrix was visualized using ComplexHeatmap.^25^ Genes that had a |log_2_(Fold Change)| > 0.5 and an adjusted *P*-value < .05 as calculated by DESeq2 analysis were input into WebGestalt for overrepresentation analysis in Reactome Pathways.^26,27^ The most significant pathways as measured by false discovery rate (FDR) were plotted as the −log_10_(FDR).

### H3K27me3 IHC

IHC was performed on FFPE tissue sections using antibodies directed against H3K27me3 (1:50 titer, clone C36B11, Cell Signaling Technology). Staining was performed on a Benchmark Ultra Immunostainer (Roche Tissue Diagnostics) and detected with OptiView DAB (Roche) after pretreatment with CC1 (Roche). H3K27me3 expression was scored as (1) retained, (2) partial loss, or (3) complete loss based on independent evaluation by two neuropathologists (A.R. and R.A.V.) and consensus review of discordant cases.

### Statistical Analysis

Patient characteristics were summarized with frequencies and percentages or medians and ranges, as appropriate. Recurrence-free survival (RFS) was defined as the time from original diagnosis to earliest radiographic evidence of tumor recurrence (defined as the presence of new nodular enhancement in or adjacent to the resection bed after radiographically confirmed GTR), and patients were censored at date of last follow-up or death if they did not have a recurrence. Associations with RFS were assessed with likelihood ratio tests from Cox proportional hazards regression models, and hazard ratios together with 95% confidence intervals were reported. RFS at 5 and 10 years were estimated with the Kaplan-Meier method. The concordance index (c-index) was calculated as a measure of how well each predictor discriminates between those with versus without recurrence. Due to the low number of events (recurrences), no multivariable analysis was performed and no adjustment for multiple testing was performed. *P*-values less than .05 were considered statistically significant. Analyses were conducted using SAS version 9.4 (SAS Institute Inc.) or R.^21^

## Results

### Patient Characteristics

We evaluated a retrospective cohort of 63 patients who underwent radiographically confirmed GTR of an atypical (CNS WHO grade 2) meningioma at our institution between 1988 and 2011 (Table 1). The impact of radiation therapy on recurrence and survival across this cohort has been reported previously.^14^ Median age at diagnosis was 59.8 years (range 27.4–90.6) and the cohort included 41 (65%) female and 22 (35%) male patients. Tumors were located to the convexities (*n* = 45, 71%), falx/tentorium (*n* = 8), skull base (*n* = 6), sagittal sinus (*n* = 1), orbit (*n* = 1), occipital (*n* = 1), or infratentorial (*n* = 1) regions. As reported previously, 8 of 62 patients (13%) underwent fractionated radiation therapy as part of their initial treatment plan following GTR (treatment unknown for 1 patient), with a median dose of 5400 cGy (range 5051–6120) over median of 30 fractions (range 28–30).^14^ Clinical follow-up was available for all patients, with a median follow-up of 10 years (range 0.8–24.1). Tumors recurred in 17 patients, with a 5-year and 10-year RFS of 84% and 71%, respectively. There was no significant association between patient sex, tumor location, or treatment with radiation therapy and RFS. Overall, the characteristics of our patient cohort are typical of meningioma.

**Table 1.** Association of Clinical Characteristics With RFS

	*N*	Events	5-Year RFS (95% CI)	10-Year RFS (95% CI)	Hazard Ratio (95% CI)	*P*
Sex						.24
Female	41	13	81.1% (68.5, 93.8)	64.7% (48.3, 81.1)	Reference	
Male	22	4	89.8% (76.4, 100)	81.7% (62.1, 100)	0.53 (0.15, 1.50)	
Tumor location						.19
Convexity/falx/tentorium	53	13	85.7% (75.8, 95.6)	72.2% (58.4, 86.0)	Reference	
Sagittal sinus/skull base	7	1	80.0% (44.9, 100)	80.0% (44.9, 100)	0.74 (0.04, 3.75)	
Other location	3	3	66.7% (13.3, 100)	33.3% (0, 86.7)	3.71 (0.84, 11.71)	
Radiation Therapy (RT)						.66
None	54	14	83.5% (73.0, 94.0)	73.8% (60.9, 86.7)	Reference	
Fractionated RT	8	3	87.5% (64.6, 100)	52.5% (12.5, 92.5)	1.34 (0.31, 4.10)	

Abbreviations: RFS, recurrence-free survival; CI, confidence interval.

### Identification of Molecular Driver Alterations by Targeted NGS

**Figure 1.**
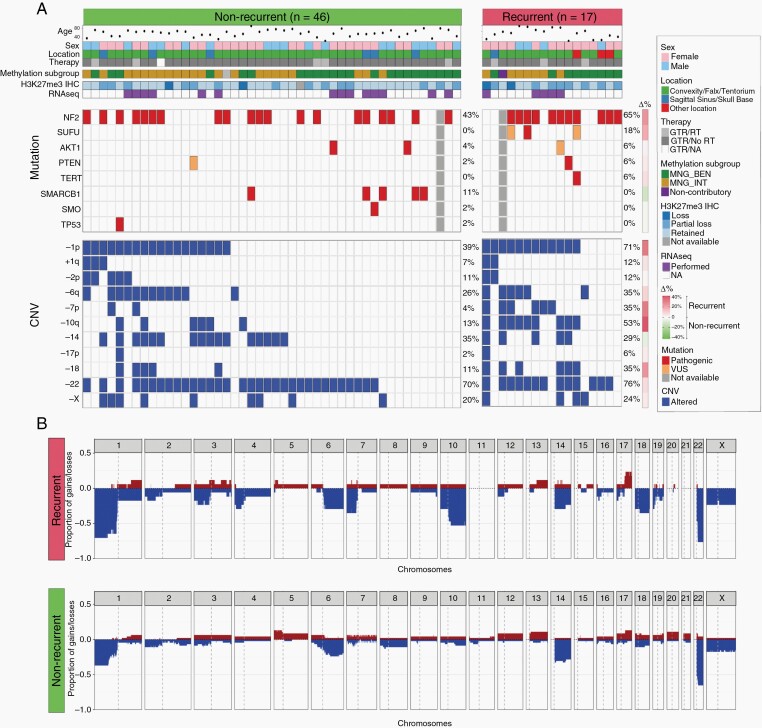
Clinical and genomic features of 63 patients who underwent gross total resection of a CNS WHO grade 2 meningioma. (A) OncoPrint Summary. Benign (MNG_BEN) or Intermediate (MNG_INT) methylation class assigned using the DKFZ Heidelberg classifier (v.12.5). Mutations were manually annotated as pathogenic or variants of uncertain significance (VUS) using American College of Medical Genetics and Genomics (ACMG) clinical criteria. Δ% denotes % incidence in recurrent—% incidence in nonrecurrent cases. GTR, gross total resection; RT, radiation therapy. (B) Frequency of genome wide copy number variants (CNV) in recurrent versus nonrecurrent cases.

Targeted NGS was performed in 61 tumors using a Clinical Laboratory Improvement Amendments (CLIA)-certified NGS panel covering sequence variants in 50 genes associated with CNS tumors (Figure 1). Of note, this panel includes *NF2*, *SMARCB1*, *AKT1*, and *SMO*, but does not include *TRAF7* or *KLF4*. Within our cohort, pathogenic alterations of *NF2* were frequent (*n* = 31; 51%). A smaller subset of tumors harbored pathogenic alterations in *SMARCB1* (*n* = 5; 8%), most in conjunction with *NF2* mutation (4 of 5). Rare tumors showed mutations of *AKT1* (*n* = 2), *SMO* (*n* = 1), *SUFU* (*n* = 1), *TP53* (*n* = 1), and *PTEN* (*n* = 1). A single tumor harbored a *TERT* promoter mutation, an alteration associated with poor prognosis and consistent a CNS WHO grade 3 designation within the updated 2021 WHO Classification of CNS tumors.^2^ This patient remains alive, with multiple tumor recurrences 15 years after the initial diagnosis.

**Figure 2.**
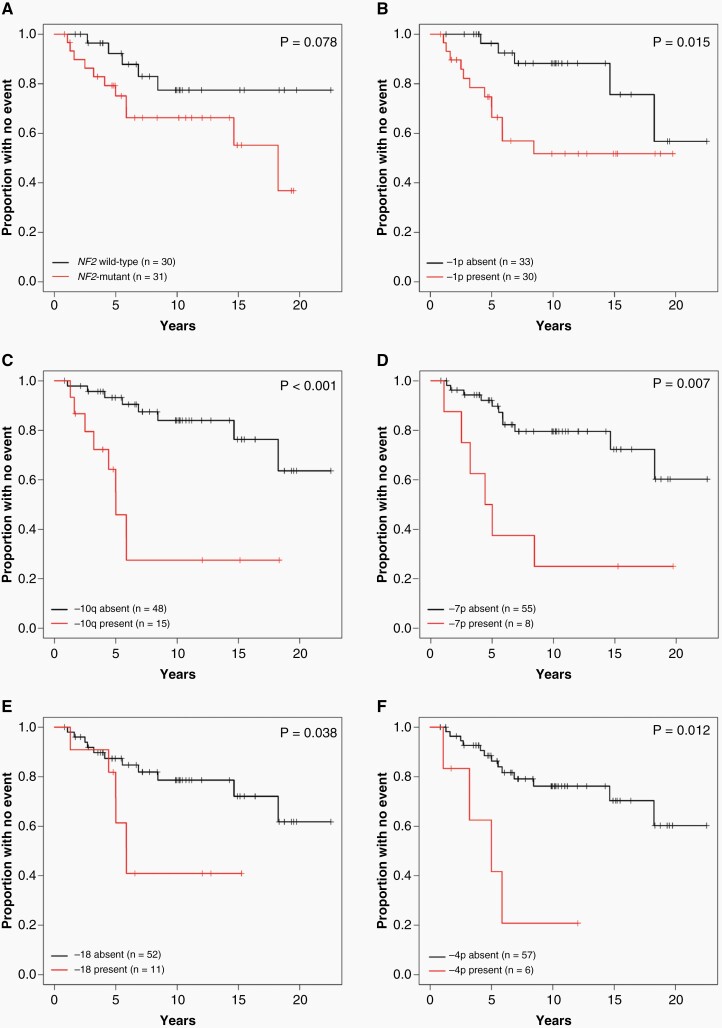
Impact of genomic alterations on RFS. Kaplan Meier curves denoting RFS of patients with tumors harboring (A) *NF2* mutation, (B) loss -1p, (C), loss -10q, (D) loss -7p, (E) loss -18 or (F) loss -4p. RFS, recurrence-free survival.

Neither *NF2* nor *SMARCB1* mutation showed statistically significant association with RFS (Table 2; Figure 2A). However, *NF2*-mutant tumors showed an apparent trend toward decreased RFS (5-year and 10-year RFS: 75% and 66% vs 92% and 77%, respectively; *P* = .08). None of the 5 tumors harboring *SMARCB1* mutation recurred, although no statistically significant association with RFS (*P* = .089) was identified in this limited subset. Overall, within our series, mutation status was not a strong predictor of progression-free survival (PFS).

**Table 2.** Association of Genomic Features With RFS

	N	Events	5-Year RFS (95% CI)	10-Year RFS (95% CI)	Hazard Ratio (95% CI)	*P*
*NF2* mutation						.08
Absent	30	5	92.2% (81.9, 100)	77.4% (59.7, 95.2)	Reference	
Present	31	11	75.1% (59.0, 91.2)	66.3% (48.0, 84.5)	2.49 (0.90, 7.91)	
*SMARCB1* mutation						.09
Negative	56	16	82.1% (71.5, 92.8)	69.2% (55.4, 83.0)	Reference	
Positive	5	0	100.0% (Non-est^a^)	100.0% (Non-est^a^)	Non-est^a^	
Methylation class (highest score)						.96
Benign	32	9	86.9% (74.9, 98.9)	75.4% (59.4, 91.4)	Reference	
Intermediate	29	7	79.7% (63.6, 95.7)	68.5% (48.4, 88.5)	1.02 (0.36, 2.76)	
Methylation class (score ≥ 0.85)						.56
Benign	16	4	85.9% (67.7, 100)	78.1% (56.0, 100)	Reference	
Intermediate	17	5	73.3% (51.0, 95.7)	62.9% (35.9, 89.9)	1.49 (0.39, 6.04)	
H3K27me3 IHC						.31
Retained	36	7	90.3% (79.8, 100)	74.5% (57.8, 91.1)	Reference	
Partial loss	22	8	81.0% (64.2, 97.7)	68.9% (47.9, 89.9)	1.84 (0.65, 5.27)	
Loss	4	2	37.5% (0, 93.6)	37.5% (0, 93.6)	3.06 (0.45, 13.10)	
Loss 1p						**.02**
Absent	33	5	96.3% (89.2, 100)	88.2% (75.7, 100)	Reference	
Present	30	12	70.6% (53.3, 87.8)	51.7% (31.4, 72.1)	3.41 (1.26, 10.75)	
Gain 1q						.20
Absent	58	15	86.9% (77.9, 96.0)	72.9% (60.0, 85.8)	Reference	
Present	5	2	50.0% (1.0, 99.0)	0.0% (Non-est^a^)	3.11 (0.48, 11.51)	
Loss 2p						.88
Absent	56	15	86.2% (76.6, 95.7)	71.4% (57.9, 84.8)	Reference	
Present	7	2	71.4% (38.0, 100)	71.4% (38.0, 100)	1.12 (0.18, 3.99)	
Loss 3p						.10
Absent	56	13	86.1% (76.5, 95.7)	76.4% (64.0, 88.8)	Reference	
Present	7	4	71.4% (38.0, 100)	35.7% (0, 74.5)	2.84 (0.79, 8.32)	
Loss 4p						**.01**
Absent	57	13	88.5% (79.9, 97.2)	76.2% (63.6, 88.8)	Reference	
Present	6	4	41.7% (0.0, 85.1)	20.8% (0.0, 57.0)	5.68 (1.56, 16.88)	
Loss 6q						.45
Absent	45	11	88.0% (78.0, 97.9)	75.7% (61.5, 89.9)	Reference	
Present	18	6	74.7% (53.0, 96.5)	58.9% (32.8, 84.9)	1.49 (0.51, 3.92)	
Loss 7p						**.007**
Absent	55	11	89.7% (81.1, 98.3)	79.5% (67.4, 91.6)	Reference	
Present	8	6	50.0% (15.4, 84.6)	25.0% (0.0, 55.0)	4.54 (1.56, 12.00)	
Loss 10q						**.0007**
Absent	48	8	93.2% (85.7, 100)	83.9% (71.9, 96.0)	Reference	
Present	15	9	55.0% (27.6, 82.4)	27.5% (1.6, 53.4)	5.72 (2.16, 15.46)	
Loss 14						.80
Absent	42	12	84.1% (72.3, 95.9)	70.2% (54.2, 86.1)	Reference	
Present	21	5	84.4% (68.2, 100)	72.8% (52.2, 93.3)	0.87 (0.28, 2.37)	
Loss 18						**.04**
Absent	52	11	87.4% (77.9, 96.9)	78.6% (65.9, 91.4)	Reference	
Present	11	6	71.6% (44.2, 99.0)	40.9% (10.4, 71.5)	3.19 (1.07, 8.69)	
Loss 22						.44
Absent	18	4	94.4% (83.9, 100)	70.9% (46.1, 95.6)	Reference	
Present	45	13	79.8% (67.2, 92.4)	70.7% (55.9, 85.5)	1.53 (0.54, 5.43)	
Loss X						.95
Absent	50	13	82.5% (71.3, 93.6)	73.8% (60.1, 87.5)	Reference	
Present	13	4	91.7% (76.0, 100)	62.9% (33.6, 92.1)	1.03 (0.29, 2.93)	

^a^Non-estimable due to low or no variability.

Abbreviations: RFS, recurrence-free survival; CI, confidence interval. P values <0.05 are in bold.

### Association of Copy Number Variants With Recurrence Risk

Copy number was assessed across the 63 meningiomas using the OncoScan chromosomal microarray platform (Figure 1 and Supplementary Figure S1). The most frequent copy number variants (CNVs) identified in our series were whole chromosome or whole arm losses, including -22 (*n* = 45; 71%), -1p (*n* = 30, 48%), -14 (*n* = 21, 33%), -6q (*n* = 18, 29%), -10q (*n* = 15, 24%), -X (*n* = 13, 21%), and -18 (*n* = 11, 18%). Apart from -22, -14, and -X, other copy number losses were found largely in conjunction with -1p (Figure 1 and Supplementary Figure S1). While chromosomal losses were more frequent than gains, 5 tumors showed a CNV profile characterized by gains of 5 or more whole chromosomes, including chromosome 5 (Supplementary Figure S1), a copy number profile that has been described in angiomatous meningioma.^28^ One patient harbored a segmental loss of one copy of *DMD* on Xp21.2, which has been associated with shorter survival in meningioma.^29^ This patient is alive without recurrence after 20 years of follow-up. No tumors harbored *CDKN2A/B* homozygous deletion.

Evaluating CNVs present in 6 or more tumors, decreased RFS was associated with -1p (*P* = .02), -7p (*P* = .007), -10q (*P* = .0007), and -18 (*P* = .04) (Table 2; Figure 2B–F). There was no significant association of -22 (*P* = .44), -14 (*P* = .80), -6q (*P* = .45), -X (*P* = 0.95), or other CNVs with RFS. Due to the limited number of recurrences in our series, multivariate analysis and correction for multiple testing were not performed. Tumors with intact 1p showed favorable RFS (5-year: 96%; 10-year: 88%) relative to tumors with -1p (5-year: 71%; 10-year: 52%). Tumors with -10q were less frequent but had a high recurrence rate of recurrence relative to tumor without 10q loss (5-year and 10-year RFS 55% and 28% vs 93% and 84%, respectively). Similarly, tumors with -7p showed a high recurrence rate relative to tumors with intact 7p (5-year and 10-year RFS 50% and 25% vs 90% and 80%, respectively). Overall, the presence of specific CNVs was strongly associated with PFS within this cohort.

### Association of Methylation Subgroups With Recurrence Risk

Genome-wide methylation profiling was performed in 62 cases and a methylation class assigned using the DKFZ methylation classifier (version 12.5). In 61 cases, the highest matching methylation class was meningioma (Supplementary Table S2A), while a single tumor grouped with medulloblastoma with a low confidence score (0.07). Using a score cutoff of ≥0.9 or ≥0.85, a definitive match was obtained in 28 (47%) and 33 cases (53%) respectively. The DKFZ classier groups meningiomas into three benign subclasses (1–3), two intermediate subclasses (A–B), one malignant subtype, and a clear cell subtype. Based on the highest score, 32 tumors (52%) matched to a benign meningioma subclass and 29 (47%) matched to an intermediate subclass, while no tumors matched to the malignant or clear cell subtypes (Supplementary Table S2A). The ratios of benign and intermediate subtypes were similar when limited to tumors with high confidence scores (≥0.85 or ≥0.9) (Supplementary Table S2A).

**Figure 3.**
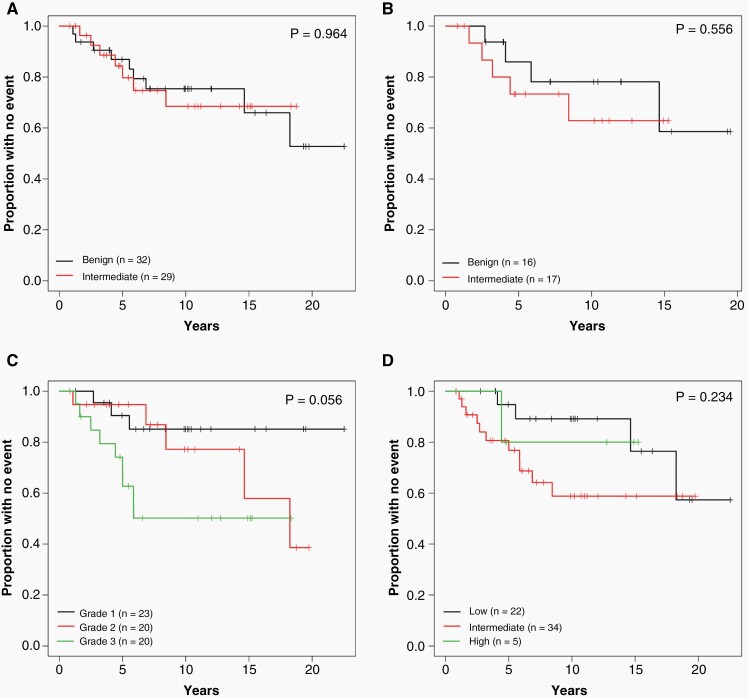
Impact of methylation class and integrated meningioma molecular classification systems on RFS. Kaplan Meier curves denoting RFS of patients with tumors matching to the DKFZ benign or intermediate meningioma class based upon (A) methylation class with the highest overall score or (B) limited to tumors with a high confidence match (score of ≥0.85). Tumors were classified according to the integrated classification schemes of (C) Driver et al.^12^ or (D) Maas et al.^13^. RFS, recurrence-free survival.

There were no significant differences in RFS based on methylation subtype (Table 2 and Supplementary Table S2B; Figure 3A and B). RFS was not significantly different for benign and intermediate subtypes, as defined by the highest matching score (5-year and 10-year RFS: 87% and 75% vs 80% and 69%, respectively; *P* = .96). Similarly, no differences in PFS were observed when limiting analysis to tumors with high confidence scores of ≥0.85 (*P* = .56) or ≥0.9 (*P* = .68). Overall, methylation subtype was not significantly associated with RFS within this series.

### RNA-Sequencing Analysis

RNA sequencing (RNA-seq) was performed in a subset of 19 cases with available frozen tumor tissue, including 5 tumors (26%) that recurred. Unsupervised analysis (hierarchical clustering and UMAP analysis) did not separate tumors by recurrence or *NF2* mutation status (Supplementary Figure S2). A set of 203 genes was identified as differentially expressed in tumors which subsequently recurred (defined by adjusted *P*-value < .05), which were not found to show clear enrichment for pathways known to be relevant to tumor recurrence/progression (Supplementary Table S3; Figure S3). While multiple independent studies have identified FOXM1 upregulation in aggressive and proliferative meningiomas,^3,30–32^ no significant difference in expression of *FOXM1* or its gene targets was identified in recurrent vs. non-recurrent tumors in our cohort. None of the genes within a recently published 36-gene meningioma prognostic signature^33^ were differentially expressed within our cohort. Similarly, neither *PTTG1*, *LEPR*, nor a set of 8 additional genes previously identified as prognostically significant in meningiomas were differentially expressed.^6^ These negative findings likely reflect the limited number of cases available for analysis but also suggest that gene expression may not be a strong predictor of recurrence in atypical meningioma.

### Association of H3K27me3 Expression With RFS

Loss of H3K27 trimethylation has been shown to correlate with aggressive behavior in meningiomas and can be evaluated using H3K27me3 IHC.^34–36^ Across our cohort, H3K27me3 was evaluated in 62 tumors, with one case excluded due to lack of expression in endothelial nuclei serving as internal controls. Four tumors (7%) showed unequivocal loss of expression of H3K27me3 in tumors cells with strong internal control staining of vessels (Supplementary Figure S4A), and 36 tumors (58%) showed unequivocal retained expression across tumor cells (Supplementary Figure S4B). In 22 tumors (35%), H3K27me3 staining was heterogeneous and difficult to interpret, with loss observed in subsets of tumor cells in areas with strong internal control staining (Supplementary Figure S4C and D). Although the 4 tumors with unequivocal loss of H3k27me3 expression had a high recurrence rate (5-year and 10-year RFS of 38%), the small number of tumors with this finding was insufficient for further statistical analysis. There was no significant difference in RFS between tumors with retained or partial loss of H3K27me3 expression (Table 2). Overall, loss of H3K27me3 expression is rare in atypical meningioma.

### Evaluation of Integrated Molecular Classification Systems

We next applied two recently published integrated meningioma classification/grading systems across our dataset (Table 3). The first (Driver et al.) combines mitotic count with the presence of specific CNVs (-1p, -3p, -4p/q, -6p/q, -10p/q, -14q, -18p/q, -19p/q, *CDKN2A/B*), to assign an integrated grade of 1, 2, or 3.^12^ In our cohort, tumors were distributed among integrated grade 1 (*n* = 23, 37%), grade 2 (*n* = 20, 32%), and grade 3 (*n* = 20, 32%). Although there was a trend toward decreased RFS in grade 2 and 3 tumors (Table 3; Figure 3C), this did not reach statistical significance in our cohort (*P* = .06). We also applied the integrated grading system described by Maas et al., which combines WHO grade, DKFZ methylation class, and specific CNVs (-1p, -6q, and -14q) to identify tumors with low, intermediate, and high recurrence risk.^13^ Across our cohort, tumors were classified as low (*n* = 22, 36%), intermediate (*n* = 34, 56%), and high-risk (*n* = 5, 8%), but did not show significant association with RFS (*P* = .23) (Figure 3D).

**Table 3.** Impact of Integrated Histologic and Molecular Grading Systems RFS on CNS WHO Grade 2 Patients

	*N*	Events	5-Year RFS (95% CI)	10-Year RFS (95% CI)	Hazard Ratio (95% CI)	*P*	C-Index
Overall	63	17	84.2% (74.7, 93.7)	71.0% (58.3, 83.6)	—	—	—
Integrated Score (Driver et al.^12^)						.06	0.67
Grade 1 (score 0–1)	23	3	90.4% (77.8, 100)	85.1% (69.5, 100)	Reference		
Grade 2 (score 2–3)	20	5	94.7% (84.7, 100)	77.2% (53.6, 100)	2.11 (0.52, 10.27)		
Grade 3 (score 4+)	20	9	68.4% (47.4, 89.4)	50.2% (26.7, 73.7)	4.35 (1.29, 19.69)		
Integrated score (Maas et al.^13^)						.23	0.65
Low (score 0–2)	22	4	94.7% (84.7, 100)	89.2% (75.0, 100)	Reference		
Intermediate (3–5)	34	11	76.8% (61.6, 92.0)	58.8% (39.3, 78.3)	2.51 (0.85, 9.10)		
High (6–9)	5	1	80.0% (44.9, 100)	80.0% (44.9, 100)	1.21 (0.06, 8.30)		
Loss 1p						.02	0.70
Absent	33	5	96.3% (89.2, 100)	88.2% (75.7, 100)	Reference		
Present	30	12	70.6% (53.3, 87.8)	51.7% (31.4, 72.1)	3.41 (1.26, 10.75)		
Loss 10q						.0007	0.72
Absent	48	8	93.2% (85.7, 100)	83.9% (71.9, 96.0)	Reference		
Present	15	9	55.0% (27.6, 82.4)	27.5% (1.6, 53.4)	5.72 (2.16, 15.46)		

Abbreviations: RFS, recurrence-free survival; CI, confidence interval; CNS, central nervous system; WHO, World Health Organization.

Calculating the c-index for each predictor, loss -10q (c = 0.72) and -1p (c = 0.70) showed stronger concordance with recurrence risk than the integrated models of either Driver et al. (c = 0.67) or Maas et al. (c = 0.65) (Table 3).

## Discussion

Recent studies have significantly expanded our understanding of the molecular drivers of meningioma^37–39^ and identified genomic and epigenic features associated with histologic grade, recurrence, and progression.^4,10,12,13,35,40^ However, molecular risk-stratification of meningiomas has yet to be fully incorporated into clinical practice. The 2021 WHO Classification of CNS Tumors has integrated select molecular parameters into the grading of meningiomas, with *TERT* promoter mutation or *CDKN2A/B* homozygous deletion now sufficient for a CNS WHO grade 3 (anaplastic) designation.^2^ These alterations are present in only a small number of meningiomas and, therefore, the vast majority of meningiomas are graded solely by histology. Recent comprehensive studies continue to confirm the significance of histologic grading.^10,12,13^ However, given the variable clinical course, there remains a lack of consensus on optimal treatment of gross-totally resected atypical (WHO grade 2) meningiomas, which is currently being evaluated in an ongoing phase III clinical trial (NCT03180268/NRG-BN003). This group of patients is most likely to benefit significantly from improved prediction of recurrence risk.

In this study, we performed detailed molecular characterization, including targeted NGS, chromosomal microarray, RNA-seq, and DNA methylation profiling, across a cohort of patients who underwent GTR, confirmed by postoperative imaging studies, of a histologically grade 2 meningioma and correlated molecular alterations with long-term clinical outcomes (median follow-up: 10 years). Within our series, the presence of specific CNVs was the strongest predictor of RFS (Table 2). Loss of 1p was frequent (*n* = 30; 48%) and significantly associated with decreased RFS (Figure 2B). Chromosome 1p loss has a well-established correlation with histologic grade^41,42^ and multiple recent studies have shown a strong association with PFS across all histologic grades of meningioma.^12,13^ Losses -4p, -7p, -10q, and -18 were also found to be strongly associated with decreased RFS in our series (Table 2; Figure 2C–F), and these CNVs have been identified to be predicative in prior studies, although somewhat less consistently than -1p.^12,13^ Losses of 6q and 14 also occur in higher-grade meningiomas and have previously been associated with unfavorable PFS in some series.^12,13^ Although frequent in our cohort, -6q and -14 were not associated with RFS (Table 2). Overall, our results confirm a strong association of specific CNVs with meningioma recurrence risk.

Within our series, *NF2* was the most frequent genomic driver mutation (*n* = 31; 51%), and a smaller subset of tumors harbored alterations of *SMARCB1* (*n* = 5, most in conjunction with *NF2*), *AKT1* (*n* = 2), *SMO* (*n* = 1), and *SUFU* (*n* = 1). At the time of sequencing, our clinically validated targeted NGS panel did not include several well-known meningioma driver genes (*TRAF7*, *KLF4*, or *POLR2A*), which is a limitation to this study. *TRAF7*, *KLF4*, *AKT1*, and *SMO* alterations are mutually exclusive with *NF2* mutation, are typically found in meningiomas localized to the skull base, and are associated with a more favorable prognosis.^4,43^ In contrast, *NF2* mutations are enriched in meningiomas of the convexities/falx and in higher-grade tumors. While we found *NF2*-mutant tumors to show a trend toward decreased RFS (Table 2; Figure 2A), this did not reach statistical significance (*P* = .08), which likely reflects the overall size and limited number of recurrences in our series. None of the 5 meningiomas harboring *SMARCB1* mutation recurred, which is in keeping with the limited published outcome data available in prior studies.^4^ Only a single tumor harbored a *TERT* promoter mutation, consistent with the frequency previously reported in atypical meningiomas.^44^ These findings suggest that CNVs are stronger predictors of RFS than underlying genomic driver mutations.

Recent large-scale efforts have focused on defining meningioma subgroups based on DNA methylation profiling, with multiple independent studies describing groups with favorable, intermediate, and poor prognoses.^8–10^ A comprehensive methylation-based classification system has been developed by the DKFZ Heidelberg group based on an analysis of 497 meningiomas, which can be readily applied to individual samples using the available online classification tool.^16^ Across meningiomas of all histologic grades, methylation-based classification showed stronger association with PFS than histology alone.^13^ Applying the DKFZ classifier to our cohort, approximately 50% of tumors were classified as benign and 50% as intermediate meningioma methylation subtypes, while no cases classified as malignant (Supplementary Table S2). Somewhat surprisingly, there was no significant difference in RFS for tumors classified as benign or intermediate subtypes, whether defined by the highest score or limiting to tumors above a threshold score of ≥0.85 (Table 2; Figure 3A and B). This may reflect, in part, the size of our series. However, it likely also reflects the well-established impact of extent of resection on overall meningioma recurrence risk, which may be abrogated in our cohort as all patients underwent radiologically confirmed GTR. Within our series, RFS of patients with an intermediate methylation subtype meningioma defined by the highest score was high relative to previously published series which included patients with both gross- and subtotal resections (5-year and 10-year RFS: 80% and 69% vs ~50% and ~30%, respectively).^10,13^ Therefore, methylation class may be a stronger predictor of RFS following subtotal (vs gross total) resection, which cannot be evaluated in our cohort. It is also important to note that a significant proportion (>30%) of meningiomas within our study as well as the previously published series^10,13^ were not definitively classified based on score cutoffs (≥0.85) commonly implemented in a clinical setting. Additionally, the DKFZ classifier version 11b4, which is used by most institutions currently performing clinical methylation profiling, includes only a single meningioma methylation class and does not currently differentiate meningioma subtypes.^10,13^ Therefore, additional validation and establishment of precise score cutoffs may be needed before methylation profiling can be implemented in the routine clinical evaluation of meningiomas.

Loss of H3K27me3 expression has been associated with poor prognosis in meningiomas across histologic grades.^34–36^ Of 62 meningiomas with interpretable H3K27me3 IHC in our series, only 4 showed definitive loss of H3K27me3 expression (Supplementary Figure S4), which was insufficient for statistical analysis of RFS association. The low frequency of H3K27me3 loss in our series is in keeping with the 4.7%–13.9% rate reported in several previously published larger series.^34–36^ Additionally, H3K27me3 expression can be heterogeneous, leading to subjectivity in interpretation. Therefore, although strongly associated with decreased RFS,^34–36^ the prognostic utility of H3K27me3 appears limited to a relatively small subset of meningiomas.

Two recently published integrated meningioma classification/ grading systems combine histologic features/ WHO grade with CNV pattern alone,^12^ or CNV and methylation class^13^ to assign an integrated grade corresponding to risk of recurrence. Across our cohort, neither of the published integrated scores was significantly associated with RFS (Table 3; Figure 3C and D), which likely reflects the relatively small size of our series. However, neither showed better prediction of recurrence risk than assessment of 1p or 10q loss alone.

While molecular characterization improves classification of meningiomas, contemporary studies continue to affirm the prognostic value of histologic grading and recognize the additive value of molecular characterization layered with histology.^12,13^ How molecular classification systems should be integrated with histology to impact patient care, however, remains less clear. Our study suggests that the presence of specific CNVs may inform therapeutic decisions for gross totally resected atypical (CNS WHO grade 2) meningiomas. Specifically, our study and recent large series uniformly confirm the prognostic significance of -1p^12,13^ The favorable outcomes among meningiomas without -1p suggest conservative management may be indicated for these patients. Multiple techniques are widely available in clinical practice to assess for 1p loss, including chromosomal microarray, fluorescence in situ hybridization, and NGS. Therefore, assessment of -1p and other CNVs can be rapidly incorporated into the clinical evaluation of atypical meningiomas.

Given the lack of consensus on the clinical management of gross totally resected atypical meningiomas, molecular predictors may help guide optimal clinical management in these patients. While cost and technology availability are currently practical limitations to comprehensive molecular profiling across meningiomas of all histologic grades, our study supports CNV profiling of atypical meningiomas to improve prediction of recurrence risk following GTR, which is amenable to ready implementation using existing, clinically validated platforms.

## Supplementary Material

vdad004_suppl_Supplementary_Materials
