# Supplementary material for: Genomic markers of recurrence risk in atypical meningioma following gross total resection
Source: Neurooncol Adv. 2023 Jan 10;5(1):vdad004. doi: 10.1093/noajnl/vdad004 (PMC9950854; doi:10.1093/noajnl/vdad004)
Supplement: vdad004_suppl_Supplementary_Materials [file vdad004_suppl_supplementary_materials.docx]

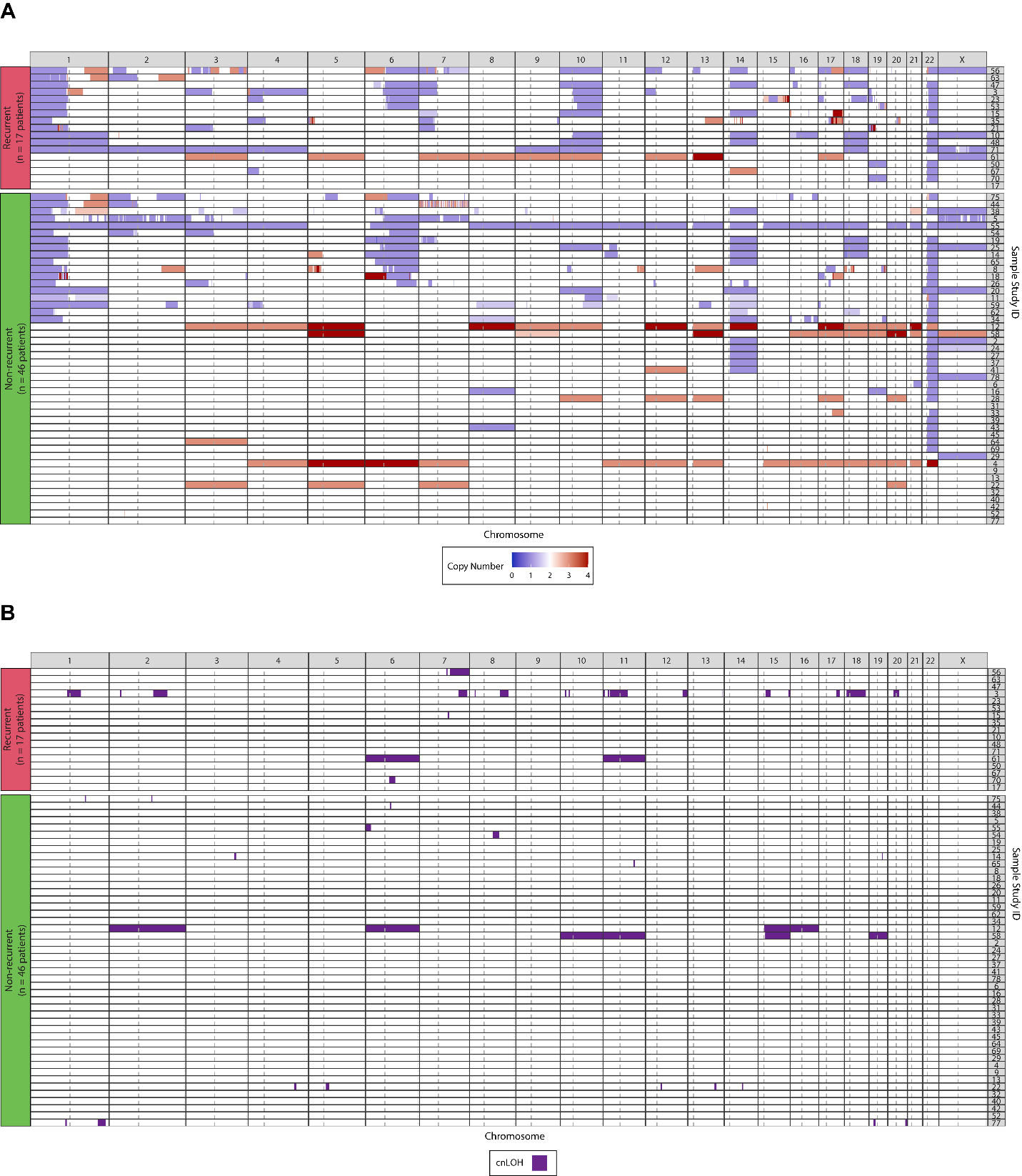


**Figure S1: (A) Genome-wide copy number gains / losses and (B) copy neutral loss of heterozygosity (cnLOH) across 63 individual tumors.** Chromosomal gains are shown in red and losses in blue.

**
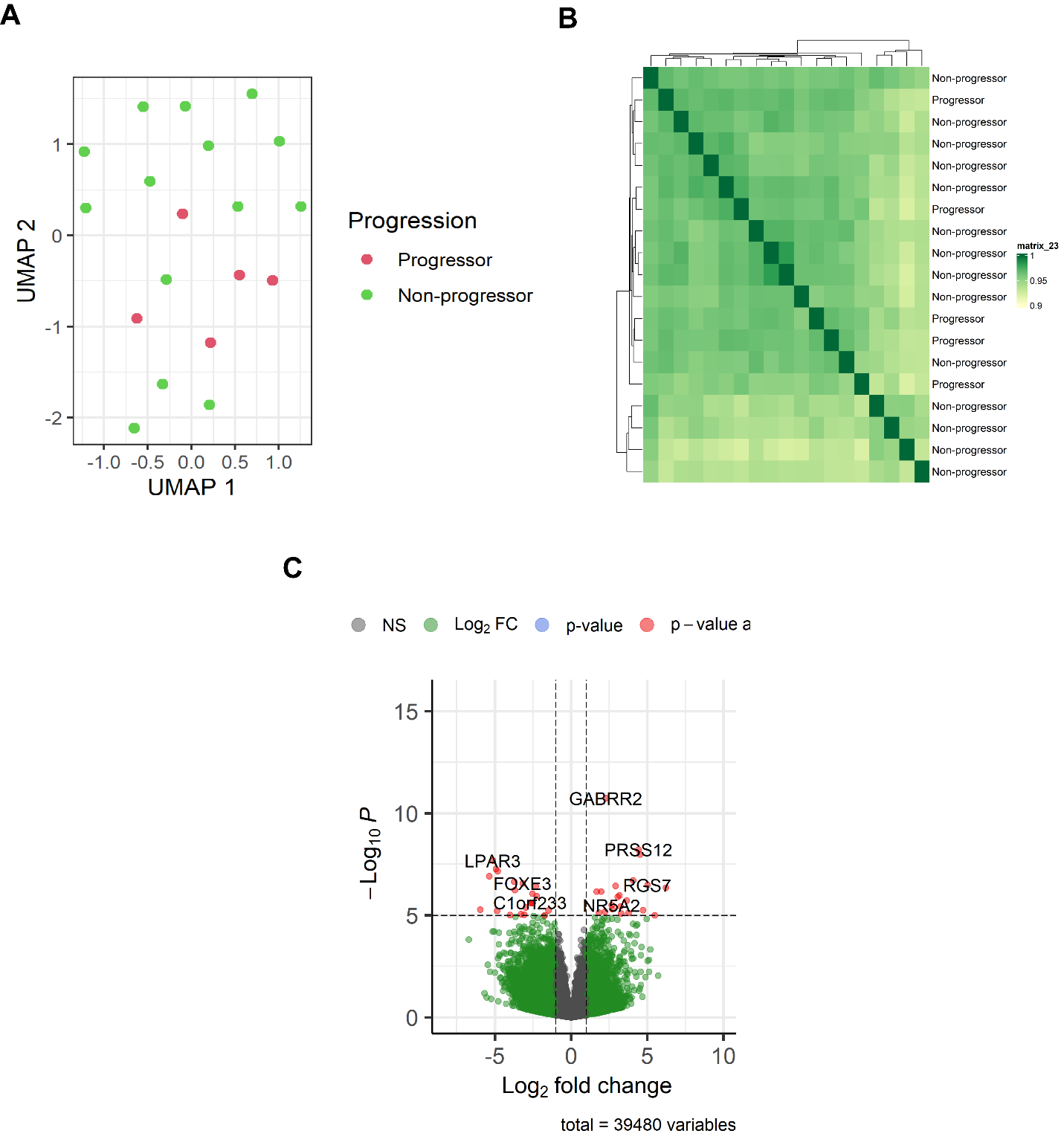
**

**Figure S2: Association of gene expression with recurrence risk.** RNA sequencing was performed on a subset of 19 atypical meningiomas with available frozen tumor tissue. (A) UMAP visualization of transcriptomic data with progressor/ recurrence status marked as fill color. (B) Volcano plot demonstrating the transcriptomic differences of meningiomas that progressed/ recurred relative to non-progressor/ non-recurrent cases. (C) Correlation matrix heatmap for the transcriptome of all 19 patients with hierarchical clustering dendrograms for the correlation matrix mapped to the top and left sides of the plot. Recurrence status is documented on the right side of the heatmap.


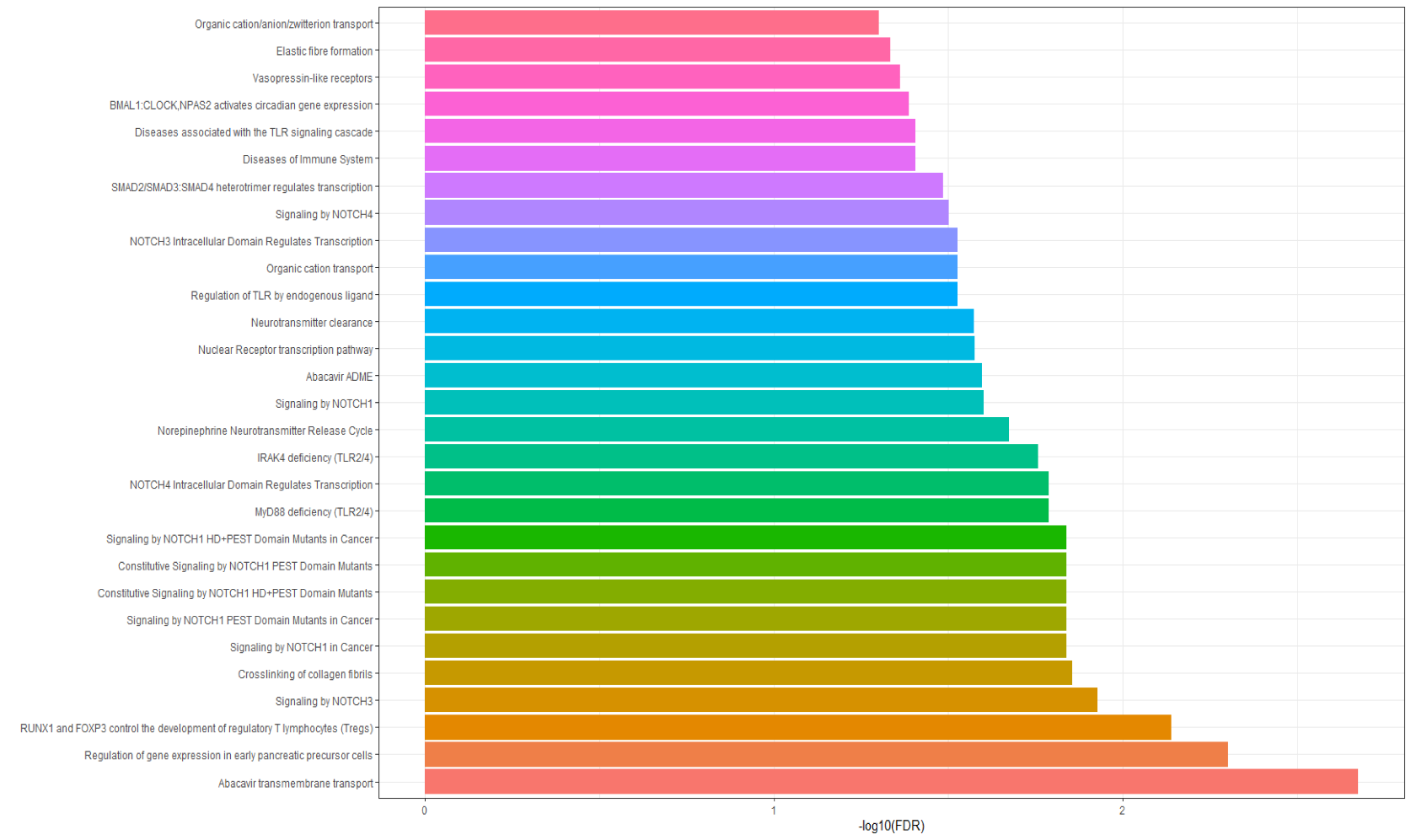


**Figure S3: Reactome pathway ORA analysis of differentially expressed genes.** Overrepresentation analysis (ORA) was performed against 203 genes differentially expressed in recurrent vs. non-recurrent cases (defined by $\left| {log}_{2}(Fold Change) \right|>0.5$ and an adjusted p-value < 0.05).


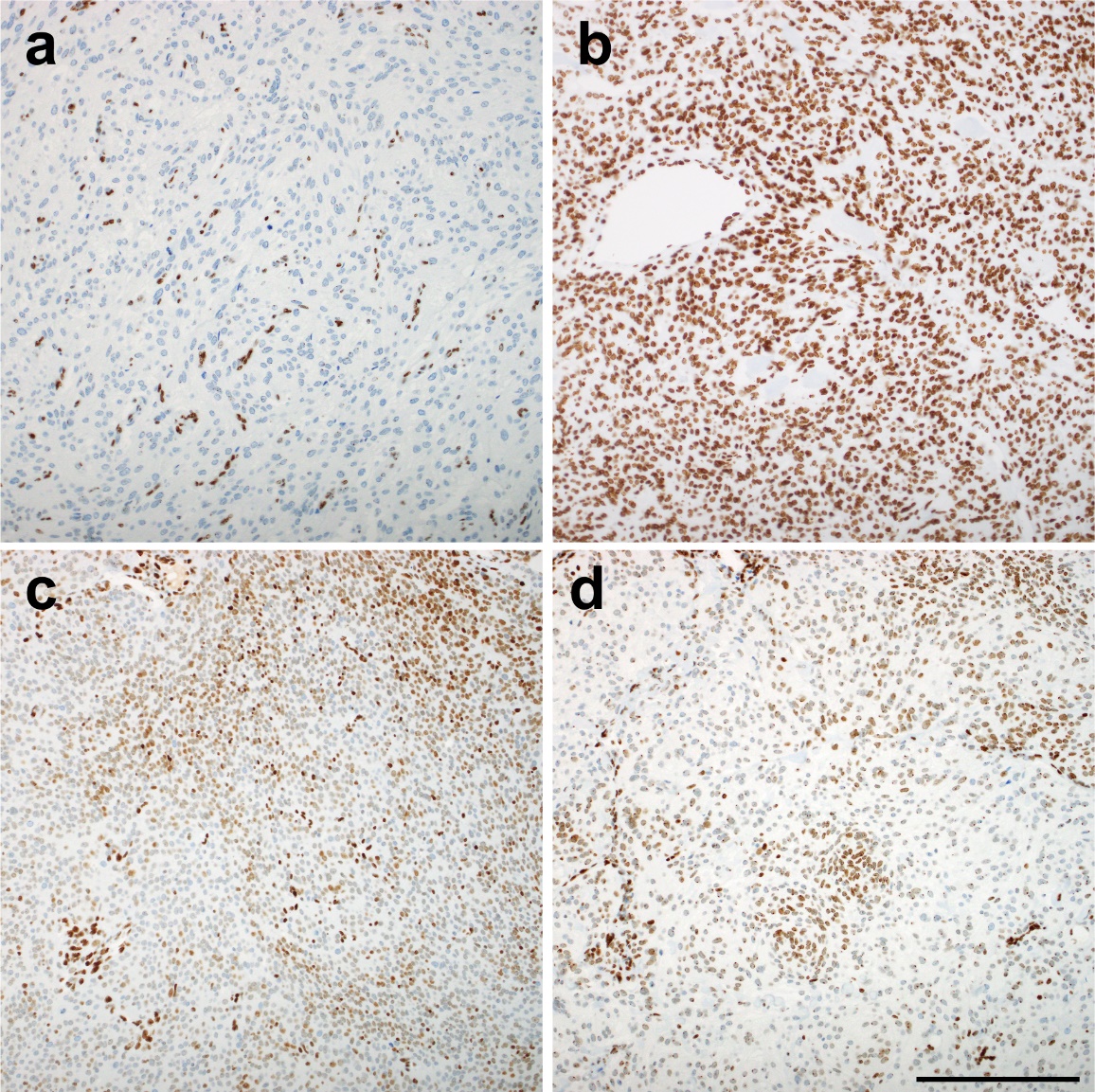


**Figure S4: H3K27me3 Immunohistochemistry.** Representative photomicrographs of atypical meningiomas with (a) unequivocal loss or (b) unequivocal retained expression of H3H27me3 by immunohistochemistry. A subset of meningiomas (c-d) showed partial loss of H3K27me3 staining with variable expression in individual cells. Scale bar = 200 µM

**Supplementary Table 1. Genes included in the custom neuro-oncology 50 gene DNA sequencing panel**

| AKT1 | FGFR2 | MYBL1 | PIK3R2 | SMO |
| --- | --- | --- | --- | --- |
| ATRX | FGFR3 | MYC | PTCH1 | STAT3 |
| BRAF | FUBP1 | MYCN | PTEN | SUFU |
| CDKN2A | GNA11 | NF1 | RB1 | TERT |
| CDKN2B | GNAQ | NF2 | SDHA | TET1 |
| CIC | GNAS | NOTCH1 | SDHB | TET2 |
| CTNNB1 | H3F3A | NOTCH2 | SDHC | TP53 |
| DAXX | IDH1 | PDGFRA | SDHD | TSC1 |
| EGFR | IDH2 | PIK3CA | SMARCA4 | TSC2 |
| FGFR1 | JAK2 | PIK3R1 | SMARCB1 | WT1 |

**Supplementary Table 2A. Subclass distribution of DKFZ benign and intermediate methylation classes (N=62).**

|  | **Highest Match** | **Score ≥0.85** | **Score ≥0.9** |
| --- | --- | --- | --- |
| **Meningioma, benign subtype** | **32 (51.6%)** | **16 (25.8%)** | **13 (21.0%)** |
| Meningioma, benign, subclass 1 | 12 | 7 | 7 |
| Meningioma, benign, subclass 2 | 7 | 3 | 3 |
| Meningioma, benign, subclass 3 | 13 | 6 | 3 |
| **Meningioma, intermediate subtype** | **29 (46.8%)** | **17 (27.4%)** | **16 (25.8%)** |
| Meningioma, intermediate, subclass A | 27 | 15 | 14 |
| Meningioma, intermediate, subclass B | 2 | 2 | 2 |
| **Medulloblastoma, Group 3** | **1 (1.6%)** | **0** | **0** |
| **No match** | **0** | **29 (46.8%)** | **33 (53.2%)** |

**Supplementary Table 2B. Association of DKFZ methylation subclass with RFS.**

|  | **N** | **Events** | **5-Year RFS (95% CI)** | **10-Year RFS (95% CI)** |
| --- | --- | --- | --- | --- |
| **Methylation Class (highest score)** | | |  |  |
| Benign, subclass 1 | 12 | 2 | 90.9% (73.9, 100) | 90.9% (73.9, 100) |
| Benign, subclass 2 | 7 | 2 | 85.7% (59.8, 100) | 68.6% (32.1, 100) |
| Benign, subclass 3 | 13 | 5 | 84.6% (65.0, 100) | 65.8% (38.2, 93.4) |
| Intermediate A | 27 | 5 | 86.7% (72.6, 100.0) | 74.5% (54.6, 94.5) |
| Intermediate B | 2 | 2 | 0.0% (Non-est^1^) | 0.0% (Non-est^1^) |
| **Methylation Class (score ≥0.85)** | | |  |  |
| Benign, subclass 1 | 7 | 2 | 83.3% (53.5, 100) | 83.3% (53.5, 100) |
| Benign, subclass 2 | 3 | 0 | 100.0% (100, 100) | 100.0% (100, 100) |
| Benign, subclass 3 | 6 | 2 | 83.3% (53.5, 100) | 55.6% (6.9, 100) |
| Intermediate A | 15 | 3 | 84.6% (65.0, 100) | 72.5% (44.9, 100) |
| Intermediate B | 2 | 2 | 0.0% (0, 0) | 0.0% (0, 0) |
| Score<0.85 | 29 | 8 | 88.9% (76.9, 100) | 71.1% (52.7, 89.5) |

Abbreviations: RFS (recurrence-free survival); CI (confidence interval)

^1^ Non-estimable due to low or no variability

**Supplementary Table 3. List of 203 genes differentially expressed in WHO grade 2 meningiomas which subsequently recurred (defined by adjusted p-value<0.05).**

|  | baseMean | log2FoldChange | lfcSE | stat | pvalue | padj |
| --- | --- | --- | --- | --- | --- | --- |
| GABRR2 | 29.5376 | 2.291877 | 0.341194 | 6.717233 | 1.85E-11 | 4.84E-07 |
| PRSS12 | 183.7689 | 4.409906 | 0.758141 | 5.816737 | 6.00E-09 | 7.85E-05 |
| TGM2 | 17338.16 | 4.520299 | 0.789871 | 5.722831 | 1.05E-08 | 9.13E-05 |
| LPAR3 | 947.0508 | -5.1383 | 0.91689 | -5.60405 | 2.09E-08 | 0.000137 |
| GSTM5 | 835.9578 | -4.92951 | 0.906686 | -5.43684 | 5.42E-08 | 0.000284 |
| RP11-58A18.2 | 46.82741 | -4.819 | 0.893034 | -5.39621 | 6.81E-08 | 0.000297 |
| HPR | 199.586 | -5.36483 | 1.013902 | -5.29127 | 1.21E-07 | 0.000454 |
| MEOX1 | 93.74225 | 4.07273 | 0.782388 | 5.205512 | 1.93E-07 | 0.000632 |
| RP11-58A18.1 | 43.19501 | -3.73222 | 0.720243 | -5.18189 | 2.20E-07 | 0.000638 |
| FOXE3 | 49.87925 | -3.16664 | 0.615674 | -5.14337 | 2.70E-07 | 0.000706 |
| RGS7 | 202.8319 | 5.015129 | 0.982066 | 5.10671 | 3.28E-07 | 0.000743 |
| SERPINE1 | 1878.839 | 2.916363 | 0.572942 | 5.090153 | 3.58E-07 | 0.000743 |
| RP11-552D4.1 | 325.1528 | -2.3276 | 0.457808 | -5.08423 | 3.69E-07 | 0.000743 |
| CCL13 | 19.91148 | 6.218775 | 1.230656 | 5.05322 | 4.34E-07 | 0.000811 |
| GRIA1 | 104.6753 | -3.69661 | 0.739208 | -5.00077 | 5.71E-07 | 0.000995 |
| GAPDHP62 | 28.7684 | 1.670948 | 0.336472 | 4.966081 | 6.83E-07 | 0.001061 |
| KIAA0922 | 736.5647 | 1.975191 | 0.397888 | 4.964183 | 6.90E-07 | 0.001061 |
| TDRD15 | 91.4701 | -2.54881 | 0.517951 | -4.92095 | 8.61E-07 | 0.001251 |
| CD209 | 277.6068 | 3.170245 | 0.649797 | 4.878825 | 1.07E-06 | 0.001469 |
| CFAP43 | 86.32465 | -2.27228 | 0.467182 | -4.86381 | 1.15E-06 | 0.001506 |
| RP1-140K8.5 | 8.188788 | 3.063557 | 0.632158 | 4.846192 | 1.26E-06 | 0.001567 |
| CR1 | 82.08402 | 3.645037 | 0.764763 | 4.766233 | 1.88E-06 | 0.002231 |
| C1orf233 | 1037.113 | -2.67473 | 0.566804 | -4.71898 | 2.37E-06 | 0.002609 |
| CCR10 | 499.8654 | -2.63559 | 0.559429 | -4.71121 | 2.46E-06 | 0.002609 |
| SNAP91 | 343.1462 | -2.52637 | 0.536544 | -4.70861 | 2.49E-06 | 0.002609 |
| NR5A2 | 87.39681 | 2.654374 | 0.569605 | 4.660025 | 3.16E-06 | 0.00318 |
| LHX6 | 55.58067 | 3.259873 | 0.703254 | 4.635415 | 3.56E-06 | 0.00345 |
| CACNA1D | 951.7817 | -2.9827 | 0.64823 | -4.6013 | 4.20E-06 | 0.003921 |
| EDN1 | 199.3442 | 2.689974 | 0.588848 | 4.568199 | 4.92E-06 | 0.004436 |
| LINC01167 | 17.79432 | -5.96603 | 1.309608 | -4.55559 | 5.22E-06 | 0.004554 |
| ZNF560 | 106.3613 | 4.715396 | 1.037998 | 4.542778 | 5.55E-06 | 0.004683 |
| KLHL35 | 359.9644 | -1.48472 | 0.327505 | -4.53343 | 5.80E-06 | 0.0047 |
| DCX | 82.26181 | -4.86054 | 1.073243 | -4.52883 | 5.93E-06 | 0.0047 |
| RP11-389C8.2 | 167.2044 | 2.225328 | 0.493765 | 4.506861 | 6.58E-06 | 0.00506 |
| CXorf36 | 517.6367 | 1.819201 | 0.405937 | 4.481485 | 7.41E-06 | 0.005538 |
| CTA-38K21.2 | 29.3693 | 3.761788 | 0.840936 | 4.473336 | 7.70E-06 | 0.005594 |
| IGDCC3 | 49.69236 | 3.282567 | 0.735788 | 4.461293 | 8.15E-06 | 0.005758 |
| RP11-463O9.2 | 65.31816 | -3.2843 | 0.739028 | -4.44408 | 8.83E-06 | 0.006074 |
| CTD-3193K9.4 | 188.2585 | -3.07382 | 0.693134 | -4.43467 | 9.22E-06 | 0.006183 |
| CTD-2015G9.2 | 210.2946 | -3.99637 | 0.903111 | -4.42511 | 9.64E-06 | 0.006185 |
| PTPRU | 19900.45 | -1.73387 | 0.392272 | -4.42006 | 9.87E-06 | 0.006185 |
| SPINK2 | 30.71879 | 5.485428 | 1.241431 | 4.418632 | 9.93E-06 | 0.006185 |
| MIR646HG | 121.786 | -2.46133 | 0.55955 | -4.39877 | 1.09E-05 | 0.006578 |
| ANO7P1 | 112.9201 | -1.78548 | 0.406236 | -4.39519 | 1.11E-05 | 0.006578 |
| LINC01141 | 44.15707 | -3.65368 | 0.833618 | -4.38292 | 1.17E-05 | 0.006805 |
| DLL4 | 452.4822 | 1.546547 | 0.354025 | 4.368472 | 1.25E-05 | 0.007113 |
| TMEM156 | 161.3724 | -2.21371 | 0.507992 | -4.35777 | 1.31E-05 | 0.007311 |
| RP11-322E11.5 | 15.64319 | 2.160566 | 0.496527 | 4.35136 | 1.35E-05 | 0.007371 |
| HOXD-AS2 | 14.05339 | 2.673232 | 0.616138 | 4.338691 | 1.43E-05 | 0.00765 |
| WTAPP1 | 12.00769 | 4.946713 | 1.143006 | 4.327809 | 1.51E-05 | 0.007865 |
| LOXL2 | 996.4657 | 2.021722 | 0.467842 | 4.321379 | 1.55E-05 | 0.007865 |
| ZNF366 | 178.407 | 2.403961 | 0.556965 | 4.316177 | 1.59E-05 | 0.007865 |
| PAX9 | 53.81647 | 3.036518 | 0.703668 | 4.315273 | 1.59E-05 | 0.007865 |
| RP1-140A9.1 | 38.13868 | -1.84593 | 0.431702 | -4.27594 | 1.90E-05 | 0.009079 |
| SUSD1 | 384.7173 | 2.045733 | 0.47851 | 4.275214 | 1.91E-05 | 0.009079 |
| DYSF | 695.7162 | 1.710612 | 0.402562 | 4.249317 | 2.14E-05 | 0.010013 |
| PADI2 | 1652.032 | -2.51666 | 0.593866 | -4.23776 | 2.26E-05 | 0.010358 |
| RP11-66B24.7 | 35.05031 | -1.57463 | 0.373369 | -4.21735 | 2.47E-05 | 0.011019 |
| MORN4 | 684.8778 | -1.07423 | 0.254795 | -4.21606 | 2.49E-05 | 0.011019 |
| OSR2 | 19.71466 | 4.072658 | 0.967549 | 4.209253 | 2.56E-05 | 0.011167 |
| AGBL1 | 64.4665 | 4.345635 | 1.037505 | 4.188544 | 2.81E-05 | 0.011977 |
| CDH13 | 547.7941 | 2.052736 | 0.490386 | 4.185963 | 2.84E-05 | 0.011977 |
| FAM91A1 | 5834.426 | 1.970306 | 0.471852 | 4.17569 | 2.97E-05 | 0.012332 |
| PRAM1 | 711.504 | -2.24725 | 0.538672 | -4.17184 | 3.02E-05 | 0.012346 |
| SFRP1 | 12437.93 | -3.41245 | 0.825417 | -4.13421 | 3.56E-05 | 0.014118 |
| PPP1R3F | 663.9345 | -1.26816 | 0.306754 | -4.13412 | 3.56E-05 | 0.014118 |
| GSTM1 | 1052.403 | -3.06779 | 0.743216 | -4.12772 | 3.66E-05 | 0.014147 |
| MIR5047 | 1873.214 | 1.543466 | 0.374011 | 4.126795 | 3.68E-05 | 0.014147 |
| TRIM67 | 110.3414 | -2.58286 | 0.626891 | -4.12011 | 3.79E-05 | 0.014352 |
| CLEC14A | 659.6409 | 1.674375 | 0.408201 | 4.101838 | 4.10E-05 | 0.015313 |
| C1orf228 | 181.7951 | -2.04238 | 0.499777 | -4.08659 | 4.38E-05 | 0.015775 |
| PCDH17 | 160.2406 | 1.686429 | 0.41289 | 4.08445 | 4.42E-05 | 0.015775 |
| KY | 26.32503 | -3.22625 | 0.790314 | -4.08224 | 4.46E-05 | 0.015775 |
| AZIN2 | 748.4501 | -1.06319 | 0.260455 | -4.08205 | 4.46E-05 | 0.015775 |
| ESAM | 907.8779 | 1.411008 | 0.34641 | 4.073228 | 4.64E-05 | 0.016167 |
| GLI1 | 1070.418 | -3.01481 | 0.742059 | -4.06277 | 4.85E-05 | 0.016686 |
| RP11-342A23.1 | 13.43716 | -2.76018 | 0.680711 | -4.05485 | 5.02E-05 | 0.017038 |
| RP11-727A23.1 | 74.945 | 0.83709 | 0.206706 | 4.049656 | 5.13E-05 | 0.017197 |
| LURAP1L | 773.9177 | 2.483335 | 0.614935 | 4.038369 | 5.38E-05 | 0.017817 |
| CTB-147C13.1 | 13.70192 | -5.14385 | 1.283009 | -4.00921 | 6.09E-05 | 0.019723 |
| RP1-244F24.1 | 58.70328 | -3.37777 | 0.842798 | -4.0078 | 6.13E-05 | 0.019723 |
| CTA-963H5.5 | 107.8608 | -1.48148 | 0.369862 | -4.00549 | 6.19E-05 | 0.019723 |
| RSPO3 | 1006.239 | -3.73023 | 0.93212 | -4.00188 | 6.28E-05 | 0.019723 |
| APOC4-APOC2 | 435.6611 | -2.49104 | 0.622873 | -3.99928 | 6.35E-05 | 0.019723 |
| ADAMTS18 | 31.36238 | 3.08042 | 0.770651 | 3.997165 | 6.41E-05 | 0.019723 |
| F2RL1 | 178.4721 | 2.319669 | 0.580874 | 3.993414 | 6.51E-05 | 0.019804 |
| APOC2 | 429.4898 | -2.55856 | 0.641393 | -3.98906 | 6.63E-05 | 0.019939 |
| LINC01166 | 35.86763 | -4.73154 | 1.189267 | -3.97854 | 6.93E-05 | 0.020606 |
| OCA2 | 125.5335 | -4.03662 | 1.015931 | -3.97332 | 7.09E-05 | 0.020709 |
| CCR9 | 161.5643 | -3.35851 | 0.845547 | -3.972 | 7.13E-05 | 0.020709 |
| FAM132A | 393.2757 | -2.15654 | 0.543459 | -3.96817 | 7.24E-05 | 0.020814 |
| RP11-274H2.3 | 9.087866 | 2.031504 | 0.513226 | 3.958301 | 7.55E-05 | 0.021457 |
| WISP1 | 890.7503 | 2.01895 | 0.510617 | 3.95394 | 7.69E-05 | 0.021617 |
| LINC01314 | 766.87 | 3.796104 | 0.961922 | 3.946373 | 7.93E-05 | 0.021853 |
| RP11-138P22.1 | 496.5438 | -0.84042 | 0.212968 | -3.94625 | 7.94E-05 | 0.021853 |
| PLVAP | 3422.229 | 1.39348 | 0.35422 | 3.933939 | 8.36E-05 | 0.022303 |
| ZFR2 | 73.34988 | -3.66966 | 0.933127 | -3.93264 | 8.40E-05 | 0.022303 |
| LINC00925 | 53.58514 | -1.42806 | 0.363321 | -3.93057 | 8.47E-05 | 0.022303 |
| RP1-66C13.4 | 228.3827 | -0.80204 | 0.204057 | -3.93049 | 8.48E-05 | 0.022303 |
| HHLA3 | 402.4082 | -1.12767 | 0.287071 | -3.92818 | 8.56E-05 | 0.022303 |
| ABHD3 | 840.0795 | 1.57677 | 0.401556 | 3.926651 | 8.61E-05 | 0.022303 |
| PRKRIRP8 | 10.84677 | 4.289883 | 1.095668 | 3.915313 | 9.03E-05 | 0.023146 |
| TOB1 | 6888.456 | 1.124238 | 0.287397 | 3.911791 | 9.16E-05 | 0.023146 |
| FAM96AP2 | 46.47361 | 3.264239 | 0.834733 | 3.910516 | 9.21E-05 | 0.023146 |
| SLC22A2 | 329.8445 | 3.876953 | 0.992235 | 3.907296 | 9.33E-05 | 0.023146 |
| GS1-44D20.1 | 60.80533 | 1.250216 | 0.320217 | 3.904279 | 9.45E-05 | 0.023146 |
| PTPRVP | 123.548 | -2.58475 | 0.662347 | -3.9024 | 9.52E-05 | 0.023146 |
| LRRC59 | 5816.354 | 1.22856 | 0.314892 | 3.901523 | 9.56E-05 | 0.023146 |
| GSG1L | 94.43107 | -3.18137 | 0.819312 | -3.88298 | 0.000103 | 0.024756 |
| CD36 | 601.6355 | 2.163592 | 0.557962 | 3.87767 | 0.000105 | 0.025072 |
| HEY1 | 970.8227 | 2.113416 | 0.545758 | 3.872439 | 0.000108 | 0.025265 |
| RP3-436N22.3 | 30.03022 | 2.654269 | 0.685607 | 3.871414 | 0.000108 | 0.025265 |
| MEIOB | 82.24847 | -1.95267 | 0.505598 | -3.8621 | 0.000112 | 0.026016 |
| RP11-64K12.10 | 47.45107 | -1.65987 | 0.430184 | -3.85852 | 0.000114 | 0.026168 |
| BCL6B | 292.765 | 1.543319 | 0.400328 | 3.855137 | 0.000116 | 0.026302 |
| OXTR | 176.3553 | 2.188895 | 0.568759 | 3.848544 | 0.000119 | 0.026787 |
| RP11-618L22.1 | 128.7506 | -2.95837 | 0.770505 | -3.83952 | 0.000123 | 0.027554 |
| TLR4 | 2921.714 | 1.858833 | 0.485106 | 3.831811 | 0.000127 | 0.027938 |
| MLXIPL | 794.0438 | -2.1191 | 0.553176 | -3.83079 | 0.000128 | 0.027938 |
| GPR114 | 297.5941 | -1.82632 | 0.476859 | -3.82989 | 0.000128 | 0.027938 |
| TREML1 | 164.2421 | -1.75215 | 0.458312 | -3.82305 | 0.000132 | 0.028488 |
| MMRN2 | 1352.898 | 1.239949 | 0.324627 | 3.819617 | 0.000134 | 0.02865 |
| HS3ST4 | 57.66993 | -3.24089 | 0.84923 | -3.81627 | 0.000135 | 0.028805 |
| AC000032.2 | 496.8776 | -2.50564 | 0.658151 | -3.8071 | 0.000141 | 0.029653 |
| ST3GAL3 | 1197.535 | -0.78652 | 0.206972 | -3.80012 | 0.000145 | 0.030089 |
| SLC22A17 | 3862.079 | -1.44233 | 0.379828 | -3.79733 | 0.000146 | 0.030089 |
| MCM8-AS1 | 11.0788 | -1.71498 | 0.451778 | -3.79608 | 0.000147 | 0.030089 |
| GFOD1 | 748.6027 | 1.731418 | 0.456342 | 3.794121 | 0.000148 | 0.030089 |
| CTD-2231E14.2 | 2602.268 | 1.252582 | 0.330375 | 3.791389 | 0.00015 | 0.030089 |
| MORN5 | 20.30443 | -2.72724 | 0.71972 | -3.7893 | 0.000151 | 0.030089 |
| LINC01291 | 51.67752 | -6.71863 | 1.773275 | -3.78882 | 0.000151 | 0.030089 |
| THBS3 | 3842.473 | -1.28778 | 0.339964 | -3.78798 | 0.000152 | 0.030089 |
| COX11 | 3195.959 | 1.12169 | 0.296395 | 3.784444 | 0.000154 | 0.03029 |
| FOXI2 | 103.413 | -3.88265 | 1.026852 | -3.78112 | 0.000156 | 0.030408 |
| ZHX1-C8orf76 | 649.3708 | 0.636972 | 0.168638 | 3.777147 | 0.000159 | 0.030408 |
| DENND6B | 841.7594 | -1.45653 | 0.385692 | -3.77642 | 0.000159 | 0.030408 |
| TOM1L1 | 2030.476 | 1.296135 | 0.343344 | 3.775038 | 0.00016 | 0.030408 |
| KCNN2 | 26.92776 | 1.800235 | 0.477089 | 3.773373 | 0.000161 | 0.030408 |
| ZNF189 | 2419.471 | 1.265036 | 0.335332 | 3.772486 | 0.000162 | 0.030408 |
| RP11-523G9.3 | 15.64696 | -2.11177 | 0.56154 | -3.76069 | 0.000169 | 0.03139 |
| CCDC144CP | 84.32842 | 2.332474 | 0.620309 | 3.760177 | 0.00017 | 0.03139 |
| FAM84A | 234.4598 | 1.457436 | 0.387697 | 3.759216 | 0.00017 | 0.03139 |
| AQPEP | 11.42196 | 1.503669 | 0.400392 | 3.75549 | 0.000173 | 0.031638 |
| URGCP | 2713.986 | -0.71694 | 0.19107 | -3.75225 | 0.000175 | 0.031677 |
| ABCA8 | 5247.173 | 1.999997 | 0.533275 | 3.750405 | 0.000177 | 0.031677 |
| GAS2L2 | 39.97002 | -3.39506 | 0.905481 | -3.74945 | 0.000177 | 0.031677 |
| FOXL1 | 1324.786 | -1.38918 | 0.370624 | -3.74822 | 0.000178 | 0.031677 |
| MCTP1 | 333.734 | 2.048982 | 0.546897 | 3.74656 | 0.000179 | 0.031677 |
| RP11-263K19.4 | 1567.59 | -1.15511 | 0.30855 | -3.74368 | 0.000181 | 0.031828 |
| SEMA6B | 412.5338 | 1.893726 | 0.50656 | 3.738404 | 0.000185 | 0.032286 |
| ECEL1 | 628.9198 | -3.638 | 0.974205 | -3.73433 | 0.000188 | 0.032596 |
| GPR56 | 2541.164 | -1.20899 | 0.324004 | -3.73141 | 0.00019 | 0.032599 |
| TPM4 | 30052.46 | 1.015382 | 0.272148 | 3.730995 | 0.000191 | 0.032599 |
| VWFP1 | 287.3872 | 1.88723 | 0.507346 | 3.719808 | 0.000199 | 0.033723 |
| TIE1 | 709.0126 | 1.618463 | 0.435168 | 3.719171 | 0.0002 | 0.033723 |
| SAT1 | 22172.61 | 0.866105 | 0.233309 | 3.71227 | 0.000205 | 0.034434 |
| RAB3IL1 | 9940.376 | -0.88864 | 0.239519 | -3.7101 | 0.000207 | 0.034509 |
| CACNA1A | 511.0074 | -2.4304 | 0.656518 | -3.70195 | 0.000214 | 0.035411 |
| RP1-117B12.4 | 602.2301 | 1.369805 | 0.3702 | 3.700172 | 0.000215 | 0.035436 |
| ENTPD2 | 876.1443 | -2.79193 | 0.755801 | -3.694 | 0.000221 | 0.036074 |
| PDLIM3 | 112.6104 | 2.14328 | 0.580629 | 3.69131 | 0.000223 | 0.036074 |
| NUDT15 | 870.4995 | 0.860652 | 0.233221 | 3.690288 | 0.000224 | 0.036074 |
| POLG2 | 889.8445 | 1.116338 | 0.302586 | 3.689326 | 0.000225 | 0.036074 |
| KRT7 | 106.4995 | 4.157159 | 1.127898 | 3.685759 | 0.000228 | 0.03636 |
| DDX5 | 51254.11 | 1.07058 | 0.290998 | 3.678997 | 0.000234 | 0.037111 |
| GCGR | 146.1361 | -3.37983 | 0.91985 | -3.67433 | 0.000238 | 0.037569 |
| PHOSPHO1 | 315.5446 | -1.53403 | 0.417692 | -3.67264 | 0.00024 | 0.037592 |
| SCG2 | 4575.169 | -3.95258 | 1.077892 | -3.66695 | 0.000245 | 0.038208 |
| SEZ6 | 101.2925 | -2.27503 | 0.620843 | -3.66441 | 0.000248 | 0.038361 |
| RNF212 | 255.0669 | -4.07519 | 1.113943 | -3.65834 | 0.000254 | 0.038944 |
| RP11-823E8.3 | 969.1223 | 1.327311 | 0.362979 | 3.65672 | 0.000255 | 0.038944 |
| RP11-95F22.1 | 13.93949 | 1.961264 | 0.536445 | 3.656043 | 0.000256 | 0.038944 |
| VMP1 | 5084.45 | 0.993238 | 0.272289 | 3.647734 | 0.000265 | 0.039992 |
| LINC00961 | 78.21235 | 1.16067 | 0.319239 | 3.635744 | 0.000277 | 0.041443 |
| WISP1-OT1 | 180.1032 | 2.000003 | 0.550502 | 3.633057 | 0.00028 | 0.041443 |
| PLD4 | 2886.968 | -1.24165 | 0.341778 | -3.6329 | 0.00028 | 0.041443 |
| KCNK10 | 75.52959 | -2.72211 | 0.749341 | -3.63267 | 0.000281 | 0.041443 |
| HOXC13 | 7.725278 | 3.706248 | 1.022813 | 3.623582 | 0.000291 | 0.042687 |
| FANK1 | 121.7288 | -1.27981 | 0.353515 | -3.62023 | 0.000294 | 0.043001 |
| MUTYH | 805.5012 | -0.97722 | 0.270597 | -3.61136 | 0.000305 | 0.044252 |
| DDX42 | 9640.032 | 0.664392 | 0.184306 | 3.604829 | 0.000312 | 0.044825 |
| FRG2C | 9.972909 | 2.443525 | 0.678091 | 3.603534 | 0.000314 | 0.044825 |
| CTA-992D9.7 | 9.838491 | -2.67497 | 0.742749 | -3.60145 | 0.000316 | 0.044825 |
| RP4-671O14.7 | 68.26345 | -2.3046 | 0.640553 | -3.59783 | 0.000321 | 0.044825 |
| RP11-152N13.5 | 243.9017 | -1.46384 | 0.406878 | -3.59773 | 0.000321 | 0.044825 |
| RP1-68D18.4 | 197.4098 | 1.6466 | 0.457686 | 3.597662 | 0.000321 | 0.044825 |
| EFCC1 | 48.62563 | 1.645367 | 0.457416 | 3.597088 | 0.000322 | 0.044825 |
| AC016735.2 | 17.07405 | -3.12626 | 0.869197 | -3.59673 | 0.000322 | 0.044825 |
| CEP152 | 789.1301 | 1.462582 | 0.407716 | 3.587256 | 0.000334 | 0.045725 |
| PRSS30P | 199.5887 | -3.10708 | 0.866323 | -3.58651 | 0.000335 | 0.045725 |
| GRIN2D | 376.2706 | -2.07712 | 0.579162 | -3.58642 | 0.000335 | 0.045725 |
| MTND6P4 | 308.6702 | -1.54376 | 0.430576 | -3.58534 | 0.000337 | 0.045725 |
| CTD-2292M16.8 | 94.24377 | -1.16728 | 0.325629 | -3.5847 | 0.000337 | 0.045725 |
| ANG | 356.263 | -1.31782 | 0.369304 | -3.56839 | 0.000359 | 0.047901 |
| LINC01116 | 15.80571 | 1.892057 | 0.530312 | 3.567817 | 0.00036 | 0.047901 |
| DYNLRB2 | 45.31346 | -1.183 | 0.331597 | -3.5676 | 0.00036 | 0.047901 |
| RP11-192H23.5 | 147.8103 | -2.02256 | 0.566991 | -3.56718 | 0.000361 | 0.047901 |
| SNHG12 | 683.0006 | -0.80057 | 0.224722 | -3.56251 | 0.000367 | 0.048515 |
| ACVRL1 | 1301.144 | 1.865978 | 0.52424 | 3.559399 | 0.000372 | 0.048846 |
| C9orf40 | 203.3352 | 0.951225 | 0.267364 | 3.557792 | 0.000374 | 0.0489 |
| LINC01135 | 22.47124 | -2.56296 | 0.721509 | -3.55222 | 0.000382 | 0.049445 |
| CASP16 | 77.12387 | -2.30005 | 0.647652 | -3.55138 | 0.000383 | 0.049445 |
| GSTM4 | 2133.557 | -1.08249 | 0.304844 | -3.55096 | 0.000384 | 0.049445 |
